# Phenotypic transition of microglia into astrocyte-like cells associated with disease onset in a model of inherited ALS

**DOI:** 10.3389/fncel.2013.00274

**Published:** 2013-12-24

**Authors:** Emiliano Trias, Pablo Díaz-Amarilla, Silvia Olivera-Bravo, Eugenia Isasi, Derek A. Drechsel, Nathan Lopez, C. Samuel Bradford, Kyle E. Ireton, Joseph S. Beckman, Luis Barbeito

**Affiliations:** ^1^Instituto de Investigaciones Biológicas Clemente EstableMontevideo, Uruguay; ^2^Department of Biochemistry and Biophysics, Oregon State UniversityCorvallis, OR, USA; ^3^Environmental Health Sciences Center, Oregon State University, CorvallisOR, USA; ^4^Linus Pauling Institute, Oregon State UniversityCorvallis, OR, USA; ^5^Institut Pasteur de MontevideoMontevideo, Uruguay

**Keywords:** microglia, astrocytes, AbA cells, ALS, phenotypic transformation, neurodegeneration

## Abstract

Microglia and reactive astrocytes accumulate in the spinal cord of rats expressing the Amyotrophic lateral sclerosis (ALS)-linked SOD1 ^G93A^ mutation. We previously reported that the rapid progression of paralysis in ALS rats is associated with the appearance of proliferative astrocyte-like cells that surround motor neurons. These cells, designated as Aberrant Astrocytes (AbA cells) because of their atypical astrocytic phenotype, exhibit high toxicity to motor neurons. However, the cellular origin of AbA cells remains unknown. Because AbA cells are labeled with the proliferation marker Ki67, we analyzed the phenotypic makers of proliferating glial cells that surround motor neurons by immunohistochemistry. The number of Ki67 ^+^AbA cells sharply increased in symptomatic rats, displaying large cell bodies with processes embracing motor neurons. Most were co-labeled with astrocytic marker GFAP concurrently with the microglial markers Iba1 and CD163. Cultures of spinal cord prepared from symptomatic SOD1 ^G93A^ rats yielded large numbers of microglia expressing Iba1, CD11b, and CD68. Cells sorted for CD11b expression by flow cytometry transformed into AbA cells within two weeks*. *During these two weeks*,* the expression of microglial markers largely disappeared, while GFAP and S100β expression increased. The phenotypic transition to AbA cells was stimulated by forskolin. These findings provide evidence for a subpopulation of proliferating microglial cells in SOD1 ^G93A^ rats that undergo a phenotypic transition into AbA cells after onset of paralysis that may promote the fulminant disease progression. These cells could be a therapeutic target for slowing paralysis progression in ALS.

## INTRODUCTION

Amyotrophic lateral sclerosis (ALS) may be considered as a paradigm of neurodegeneration involving the progressive death of upper and lower motor neurons (Cleveland and Rothstein, 2001; Boillee et al., 2006a). A consistent neuropathological feature of ALS is the extensive inflammation around motor neurons and axonal degeneration, evidenced by the accumulation of reactive astrocytes, activated microglia and lymphocytes (Engelhardt et al., 1993; Barbeito et al., 2004; Ilieva et al., 2009; Graber et al., 2010). Neuroinflammation is evident in rodent models of inherited ALS overexpressing mutant Cu/Zn superoxide dismutase (SOD1) and in ALS human patients (Gurney et al., 1994; Bruijn et al., 1997; Howland et al., 2002; McGeer and McGeer, 2002; Appel, 2009). Several studies suggest that glial cells, including astrocytes and microglia, play a pathogenic role in ALS through promoting motor neuron death and spreading paralysis after disease onset (Hall et al., 1998; Barbeito et al., 2004; Sargsyan et al., 2005; Boillee et al., 2006b; Papadeas et al., 2011). These observations suggest that therapeutics targeting the inflammatory response of glial cells could slow ALS progression.

We have recently reported the isolation of astrocytes-like glial cells with an aberrant phenotype (AbA cells) from primary spinal cord cultures of symptomatic transgenic rats expressing the SOD1^G93A^ mutation (Diaz-Amarilla et al., 2011). Isolation was based on AbA cell’s marked proliferative capacity and lack of replicative senescence. These cells secrete soluble factors that induce motor neuron death with a higher potency than neonatal transgenic astrocytes. Aberrant astrocytes only appear after disease onset in SOD1^G93A^ rats and are localized adjacent to motor neurons, suggesting a link between generation of AbA cells and the progression of paralysis. However, the origin of AbA cells remains unknown. Because AbA cells actively proliferate in the degenerating spinal cord, we hypothesized they could originate from glial progenitors with a high proliferative potential. Previous studies have identified phagocytic microglia as well as NG2^+^ glial progenitors that proliferate during the active phase of motor neuron degeneration in ALS mice and rats (Magnus et al., 2008; Kang et al., 2010; Sanagi et al., 2010). Because we have previously shown that AbA cells do not express NG2 (Diaz-Amarilla et al., 2011), we examined whether AbA cells might be derived from microglia proliferating adjacent to motoneurons. In this study, we have characterized both *in vivo* and *ex vivo* the phenotype of proliferating glial cells in symptomatic ALS rats and found evidence that neurotoxic AbA cells result from a phenotypic transition from activated microglial cells.

## MATERIALS AND METHODS

### ANIMALS

All procedures using laboratory animals were performed in accordance with the international guidelines for the use of live animals and were approved by the Institutional Animal Committee. Male hemizygous NTac:SD-TgN(SOD1^G93A^)L26H rats (Taconic), originally developed by Howland et al. (2002), were bred locally by crossing with wild-type Sprague–Dawley female rats. Male SOD1^G93A^ progenies were used for further breeding to maintain the line. Rats were housed in a centralized animal facility with a 12-h light-dark cycle with ad libitum access to food and water. Symptomatic disease onset was determined by periodic clinical examination for abnormal gait, typically expressed as subtle limping or dragging of one hind limb. Rats were killed when they reached the end stage of the disease. Both the onset of symptomatic disease (160–170 d) and lifespan (180–195 d) in our colony were delayed considerably compared with earlier reports (Howland et al., 2002). This study was carried out in strict accordance with the IIBCE Bioethics Committee’s requirements and under the current ethical regulations of the Uruguayan Law N° 18.611 for animal experimentation that follows the Guide for the Care and Use of Laboratory Animals of the National Institutes of Health (USA). All surgery was performed under 90% ketamine – 10% xylazine anesthesia, and all efforts were made to minimize suffering, discomfort or stress.

### CELL CULTURE FROM END-STAGE SYMPTOMATIC Sod1^G93A^ RATS

Microglia cells were obtained from adult spinal cord of symptomatic SOD1^G93A^ rats (175 d) according to the procedures described by Diaz-Amarilla et al. (2011) with minor modifications. Adult age-matched non-Tg rats were used as controls. Briefly, animals were killed by deeply anesthesia, and spinal cord was dissected on ice. After the meninges were removed carefully, spinal cord was chopped finely and dissociated with 0.25% trypsin in calcium-free buffer for 5 min at 37°C. Trypsin treatment was stopped by adding DMEM/10% (vol/vol) FBS in the presence of 50 μg/mL DNaseI and mechanical disaggregation by repeated pipetting. The resulting extract was passed through an 80-μm mesh to eliminate tissue debris and then was spun. The pellet was resuspended in culture medium [DMEM/10% (vol/vol) FBS, Hepes (3.6 g/L), penicillin (100 IU/mL), and streptomycin (100 μg/mL)] and then was plated in a 25-cm^2^ tissue culture flask. Because large amounts of fat hindered cell counting, the cells isolated from individual spinal cords were plated in individual culture flasks. Culture medium was removed after 24 h and then was replaced every 48 h.

### LEUCINE-METHYL ESTER TREATMENT

Leucine-Methyl Ester (Leu-OMe, Sigma) was prepared in DMEM, pH adjusted to 7.4. Cultures from transgenic symptomatic rats were treated 3 days after plated with 25 mM of Leu-OMe during 1 h. Then, the cells were washed three times with PBS and fixed with cold methanol during 5 min (n = 3).

### IMMUNOCYTOCHEMICAL STAINING OF CULTURED CELLS

Cultured cells were fixed with absolute methanol at -20°C for 5 min on ice and then were washed three times with 10 mM PBS (pH 7.4). Non-specific binding was blocked by incubating fixed cells with 5% BSA in PBS for 1 h at room temperature. Corresponding primary antibodies were diluted in blocking solution and incubated overnight at 4°C in a wet closed chamber. The primary antibodies for microglia recognition were rabbit anti-Iba1 (1:200, Abcam), rabbit anti-CD11b (1:200, Abcam), and mouse anti-CD68 (1:300, Abcam). The antibodies used for astrocyte recognition were mouse anti-GFAP (1:500, Sigma), rabbit anti-GFAP (1:500, Sigma), mouse anti-S100β(1:400, Sigma). After washing, sections were incubated in a 1,000-fold dilution of secondary antibodies conjugated to Alexa Fluor 488 and/or Alexa Fluor 546 (1:1000, Invitrogen). Antibodies were detected by confocal microscopy using a confocal Olympus FV300 microscope.

### ANALYSIS OF MICROGLIAL MARKERS EXPRESSION

After isolation of the symptomatic spinal cord, cells were plated in 35-mm dishes at 1.2 × 10^4^ cells/cm^2^. 7 days after plating, cells were fixed and stained with microglia specific markers as described above. The analysis was performed manually in using the cell counter tool of the Image J software. Values were expressed as a percentage (± SD) of the total number of DAPI^+^ nuclei. Only healthy nuclei with clearly defined limiting membranes were counted. Cell counts were performed in duplicate.

### FORSKOLIN TREATMENT

After 20 days *in vitro*, 35 mm dishes were treated with 10 μM of forskolin (FSK; Sigma) during 3 h. Then, the cells were fixed using cold methanol and stained as described above.

### FLOW CYTOMETRIC ISOLATION

After 7 days *in vitro* the cells were incubated at 37°C with 0.25% trypsin without calcium. After 5 min, the cells were harvested in DMEM/10% (vol/vol) FBS and spun at 250 × *g* for 10 min. The resultant pellet was washed three times in PBS at 37°C. After that, the cells were re-suspended in blocking solution (PBS; 5% FBS, and 1% BSA). The cells were labeled at 4°C for 15 min with mouse anti-CD11b-FITC (1:100, Abcam) and sorted using the MoFlo^TM^ XDP–Beckman Coulter. After sorting, the cells were re-plated in a 25 cm^2^ bottle with DMEM/10% FBS.

### IMMUNOHISTOCHEMICAL STAINING OF RAT SPINAL CORDS

Animals were deeply anesthetized and transcardial perfusion was performed with 0.9% saline and 4% paraformaldehyde in 0.1 M PBS (pH 7.2–7.4) at a constant flow of 1 mL/min. Fixed spinal cord was removed, post-fixed by immersion for 24 h, and then transverse sectioned serially (30–50 μm) on a vibrating microtome. Serial sections were collected in 100 mM PBS for immunohistochemistry. After citrate antigen retrieval, free-floating sections were permeabilized for 15 min at room temperature with 0.1% Triton X-100 in PBS, passed through washing buffered solutions, blocked with 5% BSA:PBS for 1 h at room temperature, and incubated overnight at 4°C in a solution of 0.1% Triton X-100 and PBS containing the primary antibodies, mouse anti-Iba1 (1:200, Abcam), mouse anti CD163 (1:100, Serotec) for microglia recognition, and rabbit anti-GFAP (1:500, Sigma) for astrocyte recognition. A rabbit anti-Ki67 (1:400, Abcam) was used as a proliferation marker. The expression of nitrotyrosine was recognized with a mouse anti-NO_2_-Tyr (1:300, Millipore) antibody. The immunoreactivity was completely blocked by pre-incubation of the primary antibody with free nitrotyrosine (10 mM). No antigen retrieval was needed to detect nitrotyrosine. After washing, sections were incubated in 1:1,000-diluted secondary antibodies conjugated to Alexa Fluor 488 and/or Alexa Fluor 546 (Invitrogen). Antibodies were detected by confocal microscopy using a confocal Olympus FV300 microscope.

### QUANTITATIVE ANALYSIS OF AbA CELLS IN THE DEGENERATING SPINAL CORD

The number of proliferating cells labeled with Ki67 and also stained for the astrocytic marker GFAP or microglial marker Iba1 was assessed by counting the respective double-positive cells in the gray matter of the lumbar cord of symptomatic or asymptomatic SOD1^G93A^ rats. Quantification was performed only in the ventral horn, comparing the cell numbers in Rexed laminae VII and IX, which display low and high density of large motor neurons, respectively. Double-positive cells were counted in a perimeter of 100 μm, surrounding motor neurons. The analysis was performed manually in 10 histological sections per animal (two different rats for each condition) using the cell counter tool of the Image J software. Values were expressed as a ratio of double-positive cells per motor neuron. The number of double-positive cells labeled with Iba1 or CD163 and GFAP assessed by counting the respective double-positive cells in the gray matter of the lumbar cord of asymptomatic and symptomatic SOD1^G93A^ rats. Quantification was performed only in the ventral horn, comparing the cell numbers in Rexed laminae VII and IX, which display low and high density of large motor neurons, respectively. Values were expressed as the number of double-positive cells per mm^2^.

## RESULTS

### GLIAL PROLIFERATION ADJACENT TO DEGENERATING MOTOR NEURONS

**FIGURE 1:**
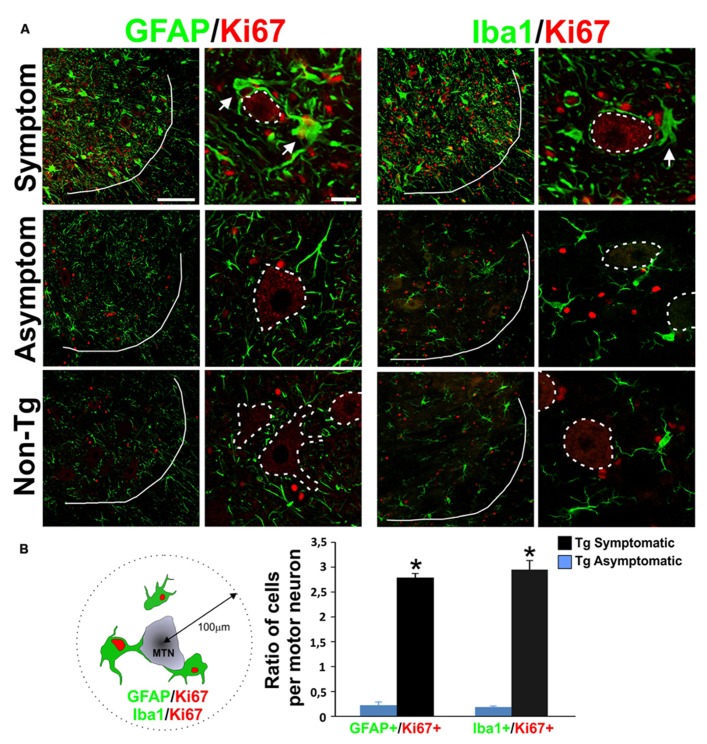
**Characterization of proliferative astrocytes and microglia in the degenerating lumbar spinal cord.** **(A)** Photomicrographs showing GFAP/Ki67 and Iba1/Ki67 stained lumbar spinal cord sections measured in symptomatic (upper panels), asymptomatic (middle panels) SOD1^G93A^ rats, and non-transgenic rats (lower panels). Low magnification panels show the notorious increase in the number of Ki67^+^ red nuclei and the appearance of large GFAP^+^ and Iba1^+^ cells (green) in the symptomatic rats, as compared to low cell proliferation in asymptomatic or non-Tg rats. A white line indicates the border between white and grey matter. The high magnification images show that GFAP/Ki67 and Iba1/Ki67 cells are typically located around the motor neurons (indicated as dotted lines) and their processes closely embrace the neuronal cell body. The arrows indicate double-labeled cells. Note the unspecific binding of Ki67 antibody to motor neuron cell bodies. **(B)** Ratio of GFAP/Ki67 and Iba1/Ki67-positive cells per motor neuron in symptomatic and asymptomatic rats. The scheme at the left shows the methods used to count the cells in a perimeter of 100 μm around motor neurons. The counting was performed in 10 histological sections per animal. Two different rats were used for each condition. Data are shown as mean ± SD; *P < 0.05. Scale bars: 100 μm for low magnification panels; 20 μm for high magnification panels.

The number of proliferating glia as identified by Ki67^+^ nuclei sharply increased in the ventral horn of SOD1^G93A^ symptomatic rats and accumulated near surviving motor neurons as well as at sites of apparent motor neuron loss (**Figure 1**). This population of Ki67^+^ cells had large cell bodies (30–50 μm) with processes embracing motor neurons and expressed GFAP and Iba1 (**Figure 1A**, upper panels). Both Ki67/GFAP and Ki67/Iba1-positive cells displayed morphological features of AbA cells in culture (Diaz-Amarilla et al., 2011) and could be easily differentiated from astrocytes and microglia from non-transgenic or Tg asymptomatic rat’s spinal cord. These large GFAP/Ki67 or Iba1/Ki67 cells were rarely observed in asymptomatic or non-transgenic rats (**Figure 1A**, lower panels). Due to the antigen retrieval procedure, motor neuron cell bodies were non-specifically labeled with Ki67 in all experimental conditions. The ratio of GFAP/Ki67 cells and Iba1/Ki67 cells to motor neurons in symptomatic rats was 2.7 and 2.9 respectively, whereas the ratio was < 0.3 in asymptomatic animals for both markers (**Figure 1B**).

### CO-EXPRESSION OF MICROGLIAL AND ASTROCYTIC MARKERS IN AbA CELLS

**FIGURE 2:**
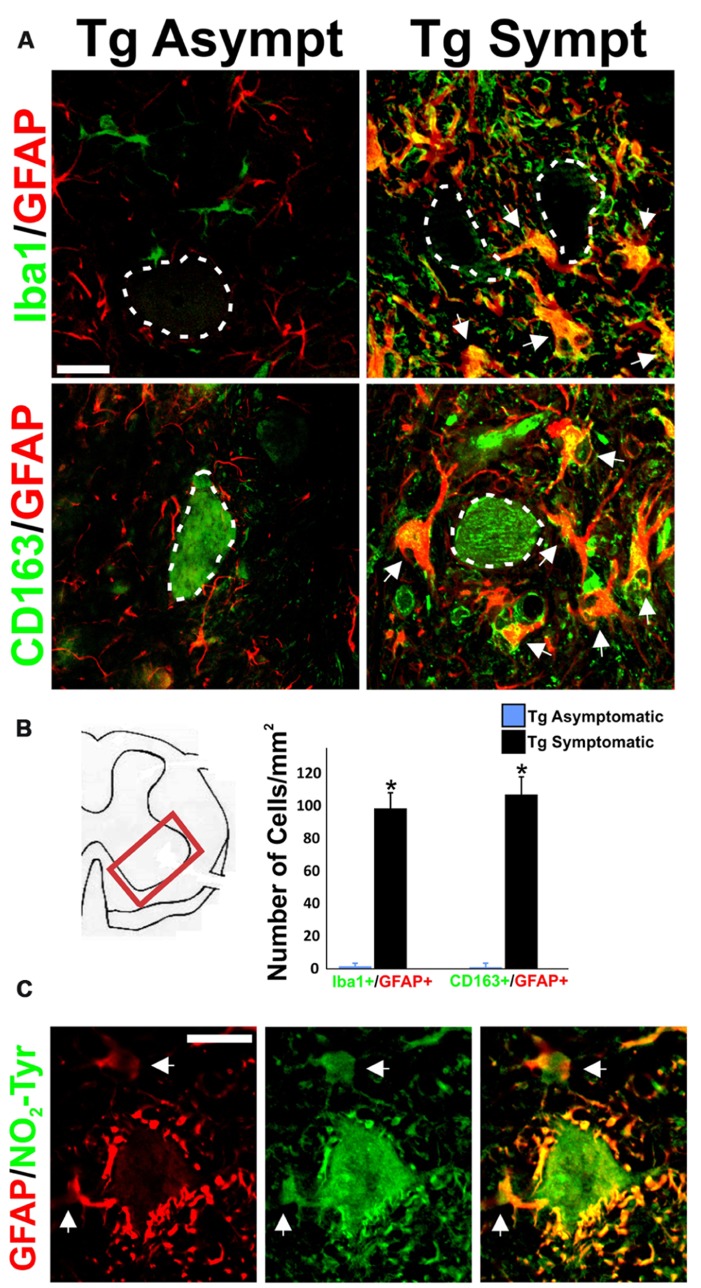
**Concurrent expression of microglia and astrocytic markers in AbA cells.** (A) Representative image of GFAP (red), Iba1 (green), and CD163 (green) in lumbar spinal cord sections from transgenic asymptomatic (Tg-asymp) and symptomatic (Tg-sympt) SOD1^G93A^ rats. Dotted lines indicate motor neuron cells bodies, which artifactually stained for CD163. Note that microglia and astrocytes in asymptomatic rat expressed GFAP and Iba1 in well-segregated cell populations. In comparison, most of the Iba1^+^ and CD163^+^ cells also expressed GFAP in symptomatic animals (arrows). **(B)** Quantification of cells expressing Iba1/GFAP and CD163/GFAP in the lumbar spinal cord. The counting was performed in 10 histological sections per animal. Two different rats were used for each condition. Data are shown as mean ± SD; *P < 0.05. **(C)** Representative confocal immunostaining against GFAP (red) and nitrotyrosine (NO_2_-Tyr, green) in the ventral horn of a symptomatic rat showing the intense nitration of Perineuronal AbA cells (arrows). Scale bars: 20 μm.

While astrocytes and microglial cells constituted two separate cell populations in asymptomatic rats, being typically detected by GFAP and Iba1 respectively, most AbA cells surrounding the motor neurons in symptomatic rats were surprisingly co-labeled with both markers as well as CD163 (**Figure 2A**). 70% of the GFAP-positive AbA cells in the ventral horn exhibited microglial markers, as compared as <1% in asymptomatic rats. The number of cells labeled with Iba1/GFAP or CD163/GFAP was similar (~100 cells per mm^2^) in symptomatic rats, suggesting the same cells expressed both microglial markers (**Figure 2B**). Detection of the microglial markers required the use of strong antigen retrieval methods, which made motor neuron to artifactually stained with CD163.

Both peri-neuronal AbA cells as well as motor neurons were strongly stained for nitrotyrosine in symptomatic rats, consistent with the production of peroxynitrite (**Figure 2C**). This immunoreactivity was completely blocked by preincubation of the primary antibody with free nitrotyrosine (data not shown).

### CHARACTERIZATION OF FIRST STAGES OF AbA CELLS *IN VITRO*

**FIGURE 3:**
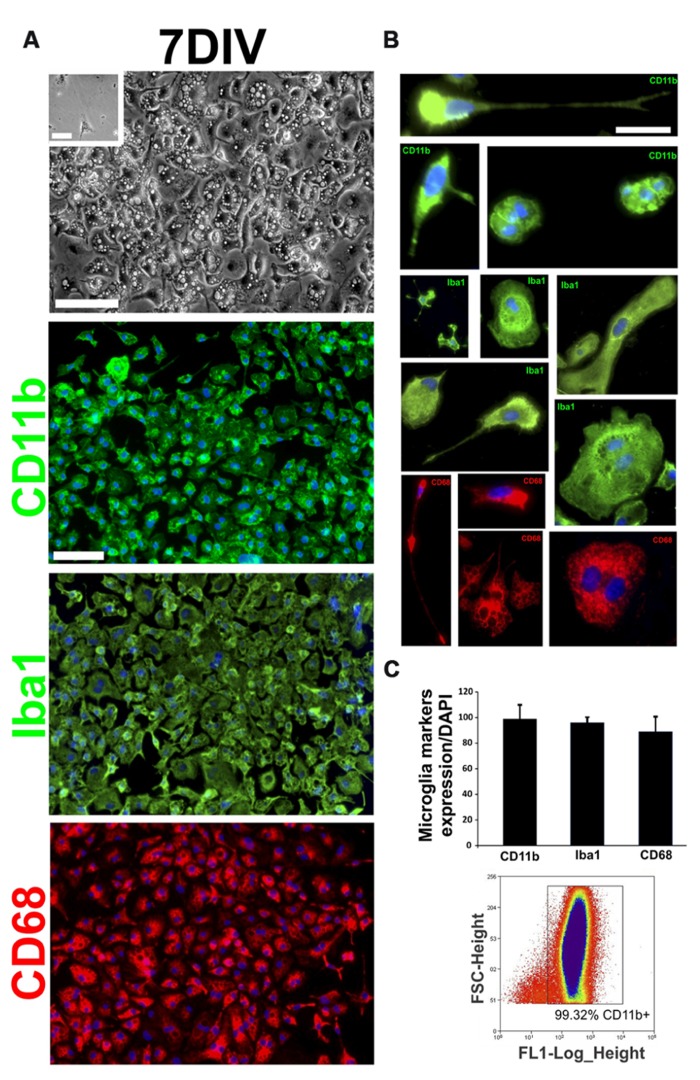
**Characterization of first stages of AbA cells *in vitro*.** Primary cultures of the spinal cord of symptomatic rats were established as in (Diaz-Amarilla et al., 2011) and the cell phenotypes were analyzed on DIV7. **(A)** The upper panel shows a representative phase-contrast image of the culture at DIV7. The inset shows the same culture obtained from non-Tg littermates where no cells could be grown. The lower panels show confocal images of cells immunostained for typical microglia markers such as CD11b, CD68, and Iba1 at 7 DIV. Scale bars: 50 μm. **(B)** Characterization of different typical microglia phenotypes present at 7 DIV, cultured from Tg symptomatic rats. Scale bar: 25 μm. **(C)** Quantification of the microglia markers expression (CD11b, CD68, and Iba1) in culture at 7 DIV, counting cell positive for microglia marker respect to the total number of nuclei labeled with DAPI (upper panel). Typical data from flow cytometry showing that 99% of cells were CD11b^+^. The data are representative of three independent experiments.

Because AbA cells can be cultured from symptomatic rats (Diaz-Amarilla et al., 2011), we determined the time course of microglial and astrocytic marker expression. Soon after primary cultures of spinal cord were established (DIV2–DIV7), most of the cells displayed the morphology of phagocytic microglia and were fluorescently labeled with the microglia markers CD11b, CD68, and Iba1 (**Figure 3A**). The detailed morphology of cultured cells closely corresponded to the typical features of microglial cells previously reported (Kreutzberg, 1996; **Figure 3B**). No immunoreactivity for GFAP or S100β was detected in cultures since the establishment of cultures and until 10–12 DIV (data not shown). Microglial cells were also analyzed by flow cytometry (FACS) using FITC-labeled CD11b antibodies. FACS analysis showed that > 99% of cells of the primary spinal cord culture of symptomatic rats belonged to the microglia lineage. The purity of this culture was also determined by counting CD68^+^ and Iba1^+^ cells and found to be > 98% (**Figure 3C**).

### PHENOTYPIC TRANSFORMATION OF MICROGLIA INTO AbA CELLS *IN VITRO*

**FIGURE 4:**
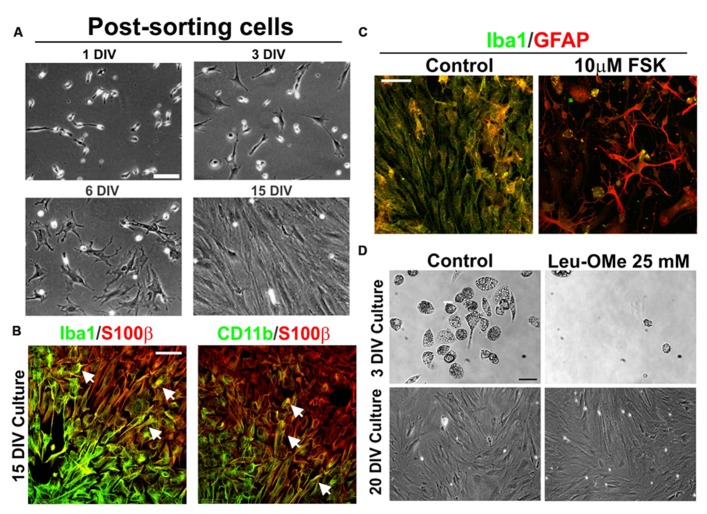
**Phenotypic transition of microglia into AbA cells in culture.** (A) Microglia cell cultures (7 DIV) from symptomatic spinal cord were dissociated and stained with FITC-labeled CD11b. After FACS sorting, cells were re-plated and analyzed for phenotypic transition at DIV15. Scale bar: 50 μm. **(B) **Confocal image showing transition zones observed at 15 DIV. Note the segregation of astrocytic S100 β staining with the microglia Iba1 or CD11b markers, which coincides with the morphological change of cells. The co-localization of astrocytic and microglia markers was found in a few cells in the border between these zones. Scale bar: 50 μ m. **(C)** Treatment of cultures at DIV15 with foskolin (10 μM, 3 h) down regulated Iba1 expression and stimulated GFAP expression and growth of processes. Scale bars: 50 μm. **(D)** Differential effect of 25 μM of Leucine-Methyl Ester (Leu-OMe) before and after the phenotypic transition. Treatment with Leu-OMe was applied to cultures at DIV3 and DIV20 to assess the toxicity. Toxicity of Leu-OMe was restricted to the microglia phenotype. This experiment was repeated with three independent isolations of AbA cells. Scale bars: 20 μm

To confirm that AbA cells were derived from microglia cell progenitors, CD11b^+^ expressing cells were FACS-sorted and re-established in culture until DIV15. Sorted cells displayed the typical microglia morphology and phenotypic marker until DIV10. This population of cells progressively transitioned into astrocyte-like cells forming monolayers between DIV10 and DIV15, while losing the morphology and phenotypic markers of microglia (**Figure 4A**). At this time, transition zones in the border separating the microglial and the astrocyte-like cells expressed both microglial (Iba1 and CD11b) and astrocytic (GFAP) markers (**Figure 4B**). These transition zones were transient because most cells expressed S100β without microglial markers by DIV15. The treatment of the cultures with 10 μM FSK, which is known to induce astrocytic processes growth and differentiation (Abe and Saito, 1997), accelerated the transition from microglia to AbA cells, while down-regulating the expression of Iba1 and promoting the growth of processes stained with GFAP (**Figure 4C**). To further confirm the microglia to astrocyte phenotypic switch, we treated the cultures with Leu-OMe, a compound used to selectively deplete microglia from primary cell cultures (Uliasz et al., 2012). **Figure 4D** shows that microglia from ALS rats were completely killed by 25 mM of Leu-OMe at DIV3, whereas there was no toxicity at DIV20 after the cells had undergone the phenotypic transition to an AbA morphology.

## DISCUSSION

Glial cells expressing mutant SOD1 are now well established to be toxic to motor neurons in rodent models as well as in ALS patients (Barbeito et al., 2004; Nagai et al., 2007; Yamanaka et al., 2008; Haidet-Phillips et al., 2011). AbA cells are the most toxic cells yet identified to motor neurons (Diaz-Amarilla et al., 2011). These distinctive glial cells are directly associated with motor neuron disease, because they actively proliferate after the onset of progressive paralysis and make intimate contact with degenerating motor neurons. By analyzing the population of proliferating Ki67^+^ glial cells in the ventral horn of symptomatic SOD1 rats, we found that AbA cells most likely originate from microglia. Notably, purified microglia isolated from the spinal cord of symptomatic rats spontaneously transformed into AbA cells.

The extremely rapid progression of paralysis in SOD1^G93A^ rats is characterized by prominent neuroinflammation associated with microglia activation (Sanagi et al., 2010; Philips and Robberecht, 2011). This inflammatory response is consistent with the sharp increase in cell proliferation others and we have observed with Ki67-staining in symptomatic animals (Schaefer et al., 2005; Pun et al., 2006). Previously, BrdU incorporation was used to demonstrate increased cell proliferation in ALS rats of NG2-positive glial progenitor cells (Magnus et al., 2008), which can potentially differentiate into astrocytes. Our results indicate that microglia more likely gave rise to AbA cells expressing astrocytic markers in regions adjacent to motor neurons.

The finding that spinal AbA cells co-express astrocytic GFAP with two different microglia markers Iba1 or CD163 was surprising because such a mixed phenotype is rarely observed. Co-expression of both markers has been observed in neoplastic glioblastoma multiforme cells (Huysentruyt et al., 2011; Persson and Englund, 2012). These human astroglial tumor cells seem to acquire phagocytic properties as a consequence of the dramatic inflammatory conditions occurring in tumors (Persson and Englund, 2012). Similarly, spinal AbA cells may originate from a phenotypic transition of inflammatory microglia into astrocytes-like cells in the degenerating cellular environment of the ventral horn. Other reports have shown aberrant features of microglial cells in symptomatic SOD1^G93A^ rats, including formation of microglia clusters (Howland et al., 2002) and multi-nucleated giant cells (Fendrick et al., 2007). Based on the morphology, localization, high proliferation rate, and other phenotypic features, spinal AbA cells are distinct from previously described M1 or M2 microglia (Kigerl et al., 2009; Durafourt et al., 2012; Liao et al., 2012). It is uncertain whether the phenotypic transition is specific for mutant SOD1 microglia or might also be observed in other CNS insults where phagocytic microglia accumulate around dying neurons (Beyer et al., 2000; Sanagi et al., 2010; Neher et al., 2012). For example, ameboid microglia-like cells expressing markers of oligodendrocyte and monocyte lineages have been described in hippocampus following acute neuronal damage (Fiedorowicz et al., 2008). AbA cells represent a novel pathological phenotype of microglia/macrophages derived from their prominent plasticity following activation (Schwartz et al., 2006; Luo and Chen, 2012). This phenotypic transformation of microglia may explain why the ablation of dividing astrocytes did not alter astrogliosis in SOD1 mice (Lepore et al., 2008).

Further evidence that AbA cells derive from activated microglia was provided in cell culture experiments showing that purified endogenous microglia can transition to astrocyte-like cells. Microglia expressing CD11b, Iba1, and CD68 represented more than 98% of cells isolated from the spinal cord of symptomatic SOD1^G93A^ rats. Moreover, FACS sorting of these cells using CD11b antibodies resulted in typical microglia cultures that also transitioned to AbA cells after 2 weeks, showing not only that AbA cells are originated from microglia but also the phenotypic change occurs *in vitro*.

Because the phenotypic switch is associated to sustained cell proliferation and a critical cell density, we suggest that inflammatory mediators secreted by the activated microglia induced the transformation. Previous reports have shown the ability of microglia to be progenitors for different neural cell types, including astrocytes *in vitro* (Yokoyama et al., 2004). This property in turn may be related to their hematopoietic origin (Yokoyama et al., 2006). Compared to AbA cells growing in the degenerating spinal cord, the concurrent expression of astrocytic and microglia markers by cultured AbA cells is only transiently in restricted to the borders of the transition zones. Therefore, it appears that activated microglial cells in degenerating spinal cord are prone to transition to astrocyte-like phenotype both in culture conditions as well as *in vivo*, in the cellular niche surrounding the motor neurons. These cells may play a role in the killing and subsequent phagocytosis of motor neurons.

Spinal AbA cells also show a number of aberrant features including high levels of S100β and Cx43 expression (Diaz-Amarilla et al., 2011) that may be relevant for neuronal toxicity through secreted S100 proteins as well as extracellular ATP released through connexin hemichannels. Activation of the extracellular ATP receptor/channel P2X7 has recently been shown to induce motor neuron death and to induce the neurotoxic phenotypes of astrocytes in culture (Gandelman et al., 2010, 2013). Furthermore, endogenous nitration of tyrosine near the ATP binding pocket of HSP90 activates P2X7, which induces motor neuron apoptosis (Franco et al., 2013).

We also showed that spinal AbA cells are strongly stained for nitrotyrosine especially in the distal perineuronal processes, consistent with the production of peroxynitrite. Microglia bearing mutant SOD1 has been shown to damage motor neurons through the production of peroxynitrite (Thonhoff et al., 2012).Because their microglia origin, spinal AbA cells may have primed to generate superoxide and hence peroxynitrite on the exterior face of the plasma membrane in close proximity to motor neurons (Beckman et al., 2001).

## CONCLUSION

Taken together, the present work supports the concept that aberrant astrocyte-like cells in the degenerating spinal cord are derived from activated microglia that proliferate around damaged motor neurons. The present study provides evidence that microglia isolated from the spinal cord of ALS–SOD rats developing paralysis have the potential to transition into an astrocyte-like phenotype. The proliferating spinal AbA cells concurrently express markers of both microglia and astrocytes lineages. Because the appearance of AbA cells is closely associated to the progression of paralysis in SOD1^G93A^ rats, a better understanding of the mediators inducing the phenotypic transition may provide another avenue of intervention to slow the progressive spread of disease in ALS patients.

## AUTHOR CONTRIBUTIONS

Emiliano Trias, Pablo Díaz-Amarilla, Silvia Olivera-Bravo, Joseph S. Beckman, and Luis Barbeito designed research; Emiliano Trias, Pablo Díaz-Amarilla, Silvia Olivera-Bravo, Eugenia Isasi, Derek A. Drechsel, Nathan Lopez, C. Samuel Bradford, and Kyle E. Ireton performed research; Emiliano Trias, Pablo Díaz-Amarilla, C. Samuel Bradford, Joseph S. Beckman, and Luis Barbeito analyzed data; and Emiliano Trias, Joseph S. Beckman, and Luis Barbeito wrote the paper.

## Conflict of Interest Statement

The authors declare that the research was conducted in the absence of any commercial or financial relationships that could be construed as a potential conflict of interest.
